# An Exploratory Examination of Interpersonal Interactions between Peers in Informal Sport Play Contexts

**DOI:** 10.1371/journal.pone.0154275

**Published:** 2016-05-04

**Authors:** Karl Erickson, Jean Côté

**Affiliations:** 1 Institute for the Study of Youth Sports, Department of Kinesiology, Michigan State University, East Lansing, MI, United States of America; 2 School of Kinesiology & Health Studies, Queen’s University, Kingston, ON, Canada; Research Center for Sports Sciences, Health and Human Development (CIDESD), University of Trás-os-Montes e Alto Douro, Vila Real, Portugal, PORTUGAL

## Abstract

Athlete-driven informal sport play represents an important context for athlete development. However, in contrast to coach-driven organized sport, little is known about the interpersonal processes driving development in this context. The present study was an exploratory descriptive analysis of the interactive peer behaviors occurring in an informal sport play setting and their relationship to athlete psychological characteristics. Thirty young athletes (<25 years old, M_age_ = 19.84) participating in informal mixed-age volleyball, soccer, and basketball sessions at a community recreation center were observed and their interactive behavior coded. Participants also completed questionnaire measures of psychological characteristics (competence, confidence, character). Descriptive analyses examined the interaction patterns of young athletes in these contexts. Multiple regression analyses were then conducted to examine the relationships between peer interactive behavior and athlete psychological characteristics. Results point to the social nature of participation in informal sport play contexts and the critical relationship between athlete competence and peer interaction tendencies. This study presents an initial exploration of peer interactive behavior in informal, mixed-age sport play contexts, but continued future research is needed to better understand the developmental processes and implications of participation in these important contexts.

## Introduction

Sport, often conceptualized as a single homogenous activity (e.g., [1]), actually takes many different forms. The different characteristics of these forms and settings create the larger context for athletes’ sport experiences [2]. In turn, these context-dependent experiences influence the outcomes athletes accrue from their participation. A primary characteristic of any sport context is the interpersonal interaction occurring within it, as constrained by the organizational structure of the activity. Most previous research on interpersonal interactions in sport has focused on formal, organized sport contexts led by a coach. In contrast, informal athlete-driven contexts have received relatively little attention. This study offers an exploratory first look at the interpersonal interactions occurring in informal sport contexts and their relationship to young athletes’ psychological characteristics.

The idea that different sport contexts differentially contribute to athlete development has been addressed by a number of authors (e.g., [3, 4, 5, 6, 7]) through retrospective studies of the developmental activities of elite athletes. The results of this previous work suggested that elite athletes often participated not only in highly structured training and competition in their younger years, but also high levels of more informal sport play with friends (e.g., backyard soccer). Previous research has linked increased participation in these informal sport play contexts to both the acquisition of sport expertise [8, 9] as well as positive youth development outcomes [10]. However, we have very little understanding of the internal dynamics of these participation experiences that may influence such development.

Typically, play theorists (see reviews by [11, 12]) have defined activities as playful if participation meets three primary criteria, namely that the activity is: 1) freely chosen, 2) personally directed (the participants themselves, rather than an external authority, control the structure and form of the activity), and 3) intrinsically motivated (engaged in for the enjoyment of the activity itself, rather than for any purposeful goal). While there is some disagreement as to whether sport can truly be called play [13, 12], informal sport play activities are similarly differentiated from more organized sport activities on a number of dimensions [2]. For example, participation in informal sport play activities is by nature intrinsically motivated and focused on maximizing enjoyment of the present experience rather than long-term performance improvement. While organized sport activities can be intrinsically motivating, there is often a focus on performance or the progressive development of sport skills planned and led by a coach. Of particular importance, informal sport play activities are governed by the participants themselves, with minimal external direction (e.g., coaching). This self-governance means that participants organize the form and structure of the activity, with the flexibility to adapt and negotiate rules to fit the constraints of the group or the setting. Finally, while organized sport activities are typically age-segregated, informal sport play activities often occur in mixed-age groupings with a corresponding variety of skill levels and physical abilities [14].

These qualities of informal sport play create a unique context for interpersonal processes. The significant influence of social interaction on human development has been noted extensively in the general developmental psychology literature [15, 16]. Indeed, the kinds of interactions and degree of social participation occurring within a given setting have been posited as key differentiators of developmental contexts [17]. Within sport research, examination of these critical processes has been conducted almost exclusively in organized sport contexts. Accordingly, interactions between coaches and athletes has received by far the most research attention (see [18, 19]), with interactions between peers studied to a comparatively lesser degree [20]. By definition lacking a coach, interactions between peers are the dominant (and only) interpersonal interactions occurring in informal sport play contexts. Without a coach guiding the activity, peers in informal sport play contexts also have the added responsibility of negotiating the form and structure of their participation through their interactions. Further differentiating peer interactions in informal sport play contexts from those in organized sport contexts are the often mixed age and ability characteristics of informal sport play activities. Thus, while peers in organized sport contexts are typically similar in age and general ability (though variability obviously exists to some degree), young people in informal sport play contexts are often interacting with peers representing a much wider variation in age and ability levels. Overall, we currently know very little about the behavioral interactions between peers in sport [21, 22] and, to our knowledge, no studies have examined peer interactions in informal sport play contexts.

One possible framework for capturing psychological characteristics potentially linked to peer interaction in this unique context is the 4C’s of athlete development as described by Côté and colleagues [23]. Encompassing competence, confidence, connection, and character, the 4C’s are a sport-specific adaptation of the 5 C’s positive youth development framework developed by Lerner and colleagues [16, 24] in general developmental science. In adapting this framework to sport research, Côté and colleagues [23] collapsed the caring/compassion dimension of the original C’s with the character dimension to better reflect the overlapping conceptualization of these constructs within the sport psychology literature. Defined broadly in this capacity and for the purposes of this study, competence refers to typical notions of sport skill, confidence refers to general perceptions of self-worth and efficacy within the sport domain, connection refers to positive bonds with other people, and character refers to both moral development and empathic/compassionate understanding of others. In combination, the 4C’s represent a full spectrum of positive psychological characteristics associated with sport participation and have successfully discriminated between athletes on different longitudinal development trajectories [25].

Thus, the purpose of the present research was to provide an exploratory descriptive analysis of young athletes’ peer interactive behavior in informal sport play contexts. Further, the present study examined the relationship between these peer interactive behaviors in informal sport play contexts and athletes’ psychological characteristics as defined by the 4C’s framework. This work was intended to provide a necessary initial descriptive foundation to guide future longitudinal research in these unique but influential sport contexts. To this end, the present study was guided by two primary questions: 1) to what degree is variation in athletes’ levels of the measured psychological characteristics associated with variation in their observed peer interactive behavior? and 2) are there differences in the strength of the associations between the different psychological characteristics and peer interactive behaviors? However, given the exploratory cross-sectional nature of this study, no specific psychological characteristic-behavior association hypotheses were forwarded, nor were hypotheses of directional causality tested.

## Methods

### Participants

All study procedures were approved by the general research ethics review board (GREB) at Queen’s University and by the recreation center’s executive prior to the initiation of data collection. Informed consent was granted in writing by all participants prior to any data collection, including written consent from parents or guardians for any participants younger than 18 years old. Participants for the present study were athletes aged 25 and younger who participated in all-ages informal volleyball, basketball, or indoor soccer sessions at a community recreation center in a mid-sized Canadian city. Of the 68 athletes who agreed to participate, 44 were within the targeted age range (given the focus of the present study on young athletes). Of these, only the final sample of 30 participants provided fully analyzable data. Mean age of the final sample was 19.84 (*SD* = 2.40), including 21 males (70%) and 9 females (30%). It should be noted that, as is often typical of informal sport play contexts, the targeted young athlete participants were embedded within a mixed-age grouping (age range = 15–49) rather than segregated into a separate participation group by themselves. Additionally, these drop-in recreation sessions were open to both genders, but typically included more male than female participants (as approximately represented in the present sample).

### Procedure

Prior to data collection, the lead author attended two weeks’ of drop-in recreation sessions at the recreation center in order to distribute information regarding the study and discuss procedures with participants. Over the following three week period, eight informal drop-in recreational sport sessions were observed and video-recorded (three volleyball sessions, three soccer sessions, and two basketball sessions). Each observed session (approx. 90 minutes in length) was recorded on video with two HD camcorders, each camcorder linked to a shotgun microphone. Consenting participants completed a questionnaire packet at the beginning of the recorded session measuring the 4C’s, as well as provided identifying information (i.e., hair colour, shirt colour) so they could be identified in the video of the session.

### Measures

#### Observational data

Behavioral data for athletes were collected via systematic observation of the videotaped sessions by an independent coder with several months of structured training and supervised experience [26] of systematic observational coding, and not involved in the study design or formulation of hypotheses. The observational data coding was conducted in a continuous manner for each participant for the duration of each session (approx. 90 minutes), resulting in a stream of time series data where the activation of a particular code also indicated the end of the previous code for that participant. All behavioral coding was conducted with Noldus Observer software [27]. For the purposes of this study, focus was placed on explicitly verbal interactive behavior; thus, non-verbal communication (e.g., head nods, high fives, fist bumps, etc. unaccompanied by any verbalization) were not coded for analysis.

Given the exploratory nature of the present study and logistical difficulties inherent to recording athletes’ verbalizations in a scattered informal setting, a simple coding system was developed for the collection of interactive behavior data. This coding system was informed by previous observational coding systems designed for peer interactive behavior in sport [28] and developmental psychology [29], adapted to the constraints of the current data collection. Athletes’ verbal interactive behavior was classified within two general categories: private and public. The private category refers to interactive behaviors intended only for those directly involved in the interaction and was subdivided into two specific codes: 1) interaction with a single individual (i.e., dyadic conversation, removed from the main group activity [e.g., not including yelling at a specific individual within the larger group context]), and 2) interaction in a group of two or more other individuals (i.e., small group conversation, quiet enough as to not be readily heard beyond the immediate small group, again removed from the main group activity). Due to the inherently quieter nature of these interactions, specific content could not be reliably distinguished on the videos and thus was not coded beyond simple occurrence of this verbal behavior and the number of individuals involved. The public category refers to interactive behaviors intended for the larger group as a whole (even if directed at a specific individual–e.g., making fun of an individual for the amusement of the larger group) and was subdivided into three specific codes: 1) organizational (i.e., selecting teams, collectively negotiating which teams play next, etc. [e.g., “You guys be a team and I’ll jump in with them”; “We got next”]) 2) sport performance-related (i.e., directing teammates where to go during game play, publicly criticizing or congratulating a teammate, etc. [e.g., “Bring the ball this way, we’ve got a good match-up over here”; “Great play, guys!”]), and 3) general (including anything non-sport related, i.e., non-sport related joking, etc. [e.g., “Check out those crazy socks!”; “Does anybody know when the gym closes tonight?”]). The distinctions between private vs public and between individual vs group within the private category were made in an attempt to capture the degree of each individual’s engagement with the larger group; positioning the size of an individual’s interactive social sphere (i.e., how many people they talk to) as a potentially differentiating interactive characteristic. Similarly, the distinctions between organizational, sport performance, and general public interaction were made in attempt to distinguish between more leadership-oriented vs more performance-oriented vs more social-oriented behaviors. Non-interactive behavior (e.g., playing or resting without actively communicating with other participants) was coded as ‘engaged in play’, thus allowing for duration-based continuous coding of all behavior. As the focus of the present study was on athletes’ interactive behavior, time spent in the ‘engaged in play’ category was excluded from analyses.

Inter-rater reliability was assessed through comparison of a 10 minute segment of video coded independently by both the primary coder and the first author, with 91% agreement between the two. Further, both the primary coder and the first author independently coded a set of 10 separate clips intentionally compiled to include a wide range of different athlete behaviors, scoring 100% agreement.

#### Questionnaire data

Measurement of athlete psychological characteristics focused on the 4C’s–competence, confidence, connection, and character. The specific battery of measures chosen was based on the extensive review and recommendations of Vierimaa, Erickson, Côté, and Gilbert [30] in their work on measurement of the 4C’s in youth sport contexts. For ratings of all C’s, athletes were instructed to refer only to their current informal sport context.

Athletes’ competence was measured using a modified version of the Sport Competence Inventory developed by Vierimaa and colleagues [30], based on the work of Causgrove Dunn, Dunn, and Bayduza [31]. In the Sport Competence Inventory, sport competence is conceptualized as consisting of three elements: technical skill, tactical skill, and physical skill. Each of these three elements was self-rated by each athlete on a 5-point Likert-type scale ranging from ‘Not at all competent’ to ‘Extremely competent’. Thus, the final competence score for each athlete is calculated as the average of the self-ratings across all three elements. For the present sample, Cronbach’s alpha assessing internal reliability was .88.

Confidence was measured using the self-confidence subscale of the Revised Competitive State Anxiety-2 (CSAI-2R: [32]). The self-confidence subscale is made up of 5 items (e.g., ‘I’m confident I can meet the challenge’) that are scored on a 4-point Likert-type scale ranging from ‘Not at all’ to ‘Very much so’. As the original version of the CSAI-2R targeted state confidence, the instructions were modified for the present study in line with the recommendations of Vierimaa and colleagues [30] to target trait sport confidence instead (i.e., “indicate how you *generally* feel” rather than “indicate how you feel *right now*”). The CSAI-2R has been validated with two independent samples of athletes and a confirmatory factor analysis revealed that the self-confidence subscale demonstrates good psychometric properties with standardized path coefficients of .69 to .80 [32]. For the present sample, Cronbach’s alpha assessing internal reliability was .94.

Connection was measured via the Peer Connection Inventory developed by Vierimaa and colleagues [30], employing a sociometric nomination approach whereby each athlete nominated the three peers they enjoyed participating with in this particular sport environment the most and the three peers they enjoyed participating with the least. Unfortunately, initial examination of raw data revealed that many participants were unable to complete full nominations as they did not know the names of most of the other participants. The connection measure was therefore excluded from further analysis.

Character was measured by the Prosocial and Antisocial Behavior in Sport Scale (PABSS: [33]). The PABSS is a 20-item questionnaire assessing how often athletes engage in specific prosocial and antisocial behaviors on a 5-point Likert-type scale. Each of the subscales has shown good internal reliability in previous research (Cronbach’s alphas = .74–.86: [33]). For the present sample, Cronbach’s alpha assessing internal reliability was .78 for the prosocial dimension and .76 for the antisocial dimension. Within the present study, an overall character score was calculated for each athlete as their score on the prosocial dimension minus their score on the antisocial dimension. The decision to collapse the prosocial and antisocial dimensions into a single character score was made for both conceptual and practical reasons. Conceptually, we felt an overall score may better represent the real-world expression of character (as intended in the C’s model) rather than a more theoretically-driven hard distinction between prosocial and antisocial. Practically, collapsing character into a single dimension also allowed us to reduce the number of variables in analyses–of concern given the relatively small sample size in this exploratory study. However, this study was, to our knowledge, the first to use this collapsed score and its use will necessarily require further theoretical and psychometric testing and validation.

### Data Analysis

Behavior in each category was represented by the total duration in seconds across the entire observed sport session for which each category was coded as active (i.e., combined duration of each athlete’s expression of a specific behavior). After initial data screening, descriptive statistics and bivariate correlations between the three remaining C’s (competence, confidence, and character) and all behavioral variables were calculated. T-tests and ANOVA’s were used to compare durations spent in each of the general behavioral categories (i.e., private vs. public) and specific actively communicative behavioral codes. Three separate standard multiple regression analyses were then run, using the duration spent expressing each of the five actively communicative behaviors as predictors of competence, confidence, and character respectively. It should be noted that, given the cross-sectional nature of the present study, prediction was considered in the purely statistical rather than causal sense, and used only to examine associative relationships. Multiple regression analyses were chosen over simple correlational analyses, however, in order to allow comparisons with respect to relative strength of prediction of each IV (behaviors) for the different DVs (competence, confidence, and character)

## Results

Initial screening of all variables used in analysis revealed no univariate or multivariate (as assessed by Mahalanobis distance) outliers. All behavioral variables were moderately positively skewed and were subsequently transformed with a square root transformation prior to analysis. Transformation brought all variables to acceptably normal distributions. Two cases had not completed the items assessing character and were excluded pair-wise on an analysis-by-analysis basis. Inter-correlations between the behavioral predictor variables were within acceptable ranges (Pearson’s *r* correlations between all variables are reported in Table 1) and variance inflation factor (VIF) scores were all less than two, suggesting the absence of multicollinearity.

**Table 1:** Correlations Between C’s and Behavior Variables.

Measures	1	2	3	4	5	6	7
1. Competence	—						
2. Confidence	.80**	—					
3. Character	.14	.03	—				
4. Private–Individual	-.02	.00	-.01	—			
5. Private–Group	.28	.17	-.07	.54**	—		
6. Public–General	.21	.24	.12	.46*	.23	—	
7. Public–Organization	.56**	.42*	-.06	.45*	.41*	.48**	—
8. Public–Sport	.22	.11	-.26	.37*	.44*	.39*	.54**

*Note*.

* *p* < .05

** *p* < .01

Descriptive statistics for all variables used in analyses are presented in Table 2. The presented durations include only the actively communicative behavior categories and do not account for time spent ‘engaged in play’ (i.e., not actively communicating) that made up the remaining time in each session for each participant. Across the entire sample, athletes spent a relatively low percentage of the total session time actively communicating (*M* = 232.21 sec, *SD* = 236.36, approximately 4.3% of total session time), though there was much variability within this range. Athletes also spent significantly more time on average in private interaction (directed at either single individuals or groups; *M* = 208.18 sec, *SD* = 211.27) than any form of public interaction combined (*M* = 24.03 sec, *SD* = 41.09; *t*(29) = 8.20, *p* < .001, Cohen’s *d* = 3.05). There were also significant differences in the time athletes spent exhibiting the five specific actively communicative behaviors (*F*(4, 26) = 21.19, *p* < .001, partial η^2^ = .77). More specifically, planned pairwise posthoc comparisons revealed significant differences between all behavioral variables (at *p* < .01 for all comparisons), with the exception of the non-significant comparison between time spent in public organizational interaction and public general interaction. Private interaction with a single individual was by far the most predominant interactive behavior (*p* < .001 for comparisons with all other behaviors). Note in particular the significantly smaller mean total durations for the entire sample for the combination of the two explicitly sport-related behaviors (Public–Organization and Public–Sport) in relation to the remaining, potentially more general, social interaction behaviors (as assessed by a planned post-hoc contract; *F*(1, 29) = 68.36, *p* < .001, partial η^2^ = .70). Also note the relatively large inter-individual variability in all behavioral categories. Significant correlations were noted between competence and confidence, between competence, confidence, and public organizational interaction, and between all behavior categories except private group interaction and public general interaction.

**Table 2:** Descriptive Statistics of All Variables for Full Sample.

Measures	Mean	SD	Min	Max
Competence (out of 5)	3.47	.94	1.00	5.00
Confidence (out of 4)	3.28	.66	2.00	4.00
Character (out of 5)	1.55	.74	-.20	3.04
Private–Individual (sec)	157.26	165.54	7.04	626.42
Private–Group (sec)	50.91	78.12	.00	297.99
Public–General (sec)	10.17	21.20	.00	105.61
Public–Organization (sec)	12.34	27.17	.00	147.05
Public–Sport (sec)	1.50	2.64	.00	12.15
Private–Total (sec)	208.18 (3.9%)	211.27	7.04	705.98
Public–Total (sec)	24.03 (0.4%)	41.09	.00	197.07
All Interaction–Total (sec)	232.21 (4.3%)	236.36	7.04	900.12

*Note*. All behavior variables measured as total duration (seconds) over the observed session. Percentage values in Mean column of last three rows indicate percentage of total session time (approx. 90 mins or 5400 seconds). Character measure calculated as prosocial score minus antisocial score (both out of 5).

Collectively, the total durations spent using the five interactive behaviors significantly predicted competence scores (*F*(5,24) = 4.23, p = 0.007), accounting from slightly more than a third of the total variance in competence (*R*^*2*^ = .47, adjusted *R*^*2*^ = .36). Individually, duration of public organizational interaction was the strongest predictor (β = .69, p = 0.002, *sr*^*2*^ unique = .28), while duration of private interaction with a single individual was a significant negative predictor (β = -.49, p = 0.020, *sr*^*2*^ unique = .05). None of the remaining three behavioral variables were significant independent predictors (private interaction with group—β = .31, *ns*; public general interaction—β = .10, *ns*; public sport-related interaction—β = -.15, *ns*). Neither of the regression models to predict confidence or character was significant.

## Discussion

The present study represents an initial foray into the interpersonal interactions occurring in informal sport play contexts. This exploratory analysis of context-specific peer interactions examined: 1) the relative expression of different interactive behaviors, and 2) the association between these different interactive behaviors and athletes psychological characteristics. While further research is needed to substantiate these early findings, descriptive analyses of the relative expression of different interactive behaviors across the entire sample revealed that participants typically spent more time engaging in general social interaction behaviors than they did engaging in explicitly sport-related communication. Further, young athletes in this mixed-age informal sport play context were far more likely to interact in a private manner, not intended for the larger group as a whole, most often with only a single other individual in dyadic-type conversation. The results from the regression analyses suggest that the primary link between interactive behaviors and athlete psychological characteristics centered on athletes’ self-perceptions of competence. In particular, there was a relatively strong positive association between competence and engaging in public organizational behaviors–in other words, taking an active role in how the shared activity was to be structured and run. A negative association was also reported between competence and private interaction with single individuals, whereby individuals who typically interacted in the least public manner possible were likely to report lower perceptions of competence.

Considered in light of the intrinsically motivated nature of informal sport play and the lack of long-term performance objectives, the preliminary observation that athletes spent more time in potentially general social interaction than sport-related interaction supports previous research emphasizing the largely social motivation of young people’s sport participation [34]. For example, while not specifically tested in the present study, self-determination theory [35] posits that relatedness (i.e., positive social connections) is a basic human need, satisfaction of which contributes to more intrinsically motivated behavior (e.g., continued participation), and has been used extensively in the study of sport participation [36]. Though this notion requires further context-specific exploration, given their essentially social nature, informal sport play contexts might then be a particularly relevant avenue for future sport participation and physical activity promotion efforts.

The significant association between competence and peer experience in sport is well supported in the literature on peers [22]. For example, physical competence has been strongly linked to social acceptance [37]. The present study expands on these findings and offers initial insight into the specific manifestation of this relationship in informal sport play contexts. The athlete-driven characteristics of these contexts, where there is no coach to set the activity structure and participants themselves therefore assume this responsibility, provide unique opportunities for different interactions to emerge. Based on these initial findings, perceived competence may be the differentiating factor in terms of which athletes step into the void to fill these central roles (though the direction of causality of this relationship has yet to be explicitly tested). The growing body of literature on athlete leadership [38], while again primarily situated in organized sport contexts currently, may be particularly relevant in guiding future research in this area. However, it should be recognized that competence in this study was self-rated; thus the reported findings may be more indicative of athletes’ self-perceptions (and possible conceptual overlap with confidence) than a more objective measure of functional competence.

Of particular note in the observed informal sport play contexts is the mixed-age nature of the participating athletes. While study participants were limited to those aged 25 and younger, it is important to consider the results in light of the fact that these interaction patterns were observed in the presence of older athletes as well. For instance, the finding that these young athletes more often interacted in a private rather than public manner, while possibly highlighting the importance of direct interpersonal connection, may also reflect the social dynamics of being young in such informal mixed-age groupings. The relationship between perceived competence and public organizational communication might also be considered in this light; it is one thing to take a leadership role amongst same-age peers, perhaps quite another to take a similar role with older adults. Thus any emerging differences or similarities between interactions patterns in organized and informal sport activities also reflect this aspect of the context. As a common characteristic of informal sport play contexts [14], mixed-age interaction may be an important situational constraint influencing the developmental effects of a given participation experience and deserves further investigation.

A number of limitations to the present study should also be considered. Based on the cross-sectional nature of the present study, no assumptions of causal direction could be made. In fact, reciprocal causality is likely [39], in that peer interactions may influence the course of athlete development but individuals’ current developmental status or characteristics will almost certainly also influence their interaction patterns with peers. With respect to measures, the self-rating of competence also makes conceptual differentiation from confidence perhaps less clear than one might like, and may not be reflective of objective competence (i.e., tested functionally or rated by multiple external observers). Additionally, the non-utility of the connection measure (because participants did not know one another well enough to name and rate other participants) limited our ability to assess the full range of the 4 C’s as a comprehensive framework. The small sample size is an obvious limitation; however, it does reveal the realities of conducting field-based research in these informal sport play contexts. In a related vein, the logistical difficulties of filming in a publicly open gymnasium without overly interfering with the natural flow of participation and interaction (and the resulting limitations in recorded audio detail) constrained the behavioral coding. In particular, the inability to hear the content of private interactions necessitated coding these behaviors at a different level of specificity compared to the more readily interpretable public behaviors.

In looking to address these limitations, future research is encouraged to employ longitudinal designs, allowing for more insight into the direction of causality as well as the assessment of additional levels of complexity in the peer experience (e.g., the upward effects of peer interactions on other peer-related factors, such as friendships, social acceptance, social roles, and group dynamics). Even with the general category of informal sport play, there may be further contextual differences. For example, there may be different interaction patterns within self-selected groups (i.e., a group of friends) compared to the public drop-in recreation setting of the present study. Similarly, while the present sample did not allow for comparison by gender, it was generally representative of the typical gendered participation discrepancy in this setting (despite the drop-in recreation sessions being open to both genders). In order to better understand both the antecedents to participation as well as potential population level impacts (or discrepancies of impacts) of these contexts, future research might be well served to explore the sociological dynamics underlying these gendered participation tendencies. While the focus of the present study was limited to verbal interpersonal communication, other interactive elements may also contribute to experiences in these informal contexts. For example, without a coach dictating drills or calling plays, the degree of involvement or inclusion in gameplay (e.g., passes received, ball contacts, etc.) may be a key social participation characteristic with potentially unique explanatory utility. Finally, while the study of informal sport play contexts has the potential to contribute valuable new insight to several bodies of literature, researchers are advised to consider the unique methodological and logistical challenges of these contexts. In contrast to organized sport contexts, the very nature of their informality makes specific settings difficult to even locate. And while organized sport settings are often routinely observed by parents and members of the public, informal play settings require particular consideration in order to not unduly interrupt the natural flow of participation. In this vein, standard approaches to assessment, especially of more social dimensions (such as connection, in this case), may also need to be reconsidered. Given the undefined and inconsistent attendance from one session to another in these contexts, where these is no static team or group membership, there may be great variety in the degree to which participants know each other. Two approaches to this issue might be considered; first, to functionally assist in the identification of other participants (e.g., providing name tags), or second, to conceptualize this variability in interpersonal familiarity as a new source of data particularly germane to these informal settings, perhaps a measure of social integration (e.g., via network analysis) that might influence participants’ experiences or change over time via the dynamics of new group processes.

## What Does This Paper Add?

Overall, while further verification of findings is obviously necessary, this exploratory study provides a first look at the interactive behaviors occurring in informal sport play contexts, as well as a first systematic observation of the inner workings of these unique contexts. While studies of peer interactions in developmental psychology are occasionally criticized as being ‘contaminated’ by particular environmental influences and thus clouding the basic processes of interest [39], in the present study, the context-specificity of these interactions was the central focus of interest. With this focus, the present study is intended to stimulate future linkages between the macro-level research concerning the influence of participation in informal sport play contexts on athlete development [40] and the existing research on peers in sport that has primarily addressed organized sport contexts [22].
